# Adrenal function testing in dialysis patients – a review of the literature

**DOI:** 10.1186/s12882-021-02541-5

**Published:** 2021-11-01

**Authors:** Lara Brotzer, Manuela Nickler, Min Jeong Kim, Beat Mueller, Claudine A. Blum

**Affiliations:** 1 Department of Endocrinology, Diabetes & Metabolism, Kantonsspital Aarau, Aarau, Switzerland; 2 Departments of General Internal and Emergency Medicine, Medical University Clinic, Kantonsspital Aarau, Tellstrasse H7, CH-5001 Aarau, Switzerland; 3 Department of Nephrology, Kantonsspital Aarau, Aarau, Switzerland

**Keywords:** ACTH test, Corticotropin test, Adrenal insufficiency, Hemodialysis, Renal replacement

## Abstract

**Background:**

Secondary adrenal insufficiency is a frequent issue in patients with renal replacement therapy. There are concerns about metabolism and clearance for adrenocorticotropic hormone (ACTH) and cortisol in addition to hemoconcentration as confounding factors during hemodialysis (HD). Therefore, ACTH testing is currently performed before or in between HD sessions. This review of the literature aims to evaluate the current evidence for validity of testing for adrenal insufficiency in patients on chronic renal replacement therapy.

**Methods:**

A literature search of PubMed database for interventional and observational clinical trials was performed. Case reports and reviews were excluded. The search included all articles published until July 2020.

**Results:**

Of 218 potentially eligible articles, 16 studies involving 381 participants were included. Seven studies performed an ACTH test before HD or in between HD sessions. There was no data available regarding ACTH testing during HD. But there was evidence of decreased cortisol levels during HD as compared to afterwards. All included 16 studies measured basal cortisol, and seven studies performed an ACTH test. Seven trials had comparable data of baseline cortisol for a quantitative analysis. Standardized mean difference of overall cortisol was 0.18 nmol/l (95%CI − 0.08 to 0.44) in the case group.

**Conclusions:**

In patients undergoing renal replacement therapy, basal serum cortisol values are comparable to healthy volunteers. There is limited data on the validity of stimulated cortisol in these patients, especially during HD.

**Trial registration:**

Registration no. CRD42020199245.

**Supplementary Information:**

The online version contains supplementary material available at 10.1186/s12882-021-02541-5.

## Background

Secondary adrenal insufficiency (AI) due to long-term glucocorticoid medication in patients on hemodialysis (HD) is a diagnostic challenge, as many kidney diseases are treated with corticosteroids, and immunosuppressive therapy after renal transplantation often includes prednisolone [1–6]. This is further complicated, as AI and renal replacement treatment may both lead to the same unspecific symptoms like fatigue and orthostatic hypotonia [1, 3, 7–9].

Undetected adrenal insufficiency may be life-threatening. Therefore, testing adrenal function in HD patients, especially after glucocorticoid therapy or in chronic hypotensive patients is a common procedure [10, 11]. The adrenocorticotropic hormone (ACTH) test is a standard test for diagnosing AI. The 250 μg ACTH test is more suitable for primary AI, while the low-dose (1 μg) ACTH test is more sensitive for secondary AI [12]. Both tests are usually carried out in the morning, as the daily physiological value of baseline cortisol is highest at this point [13]. Importantly, peak cortisol values after ACTH stimulation are not dependent of diurnal rhythms.

For patients on HD, who usually spend three half-days per week in the hospital or in community-based centers, additional appointments and time-consuming tests are cumbersome. Furthermore, finding an accessible suitable additional vein for venous puncture is often difficult in these patients. Therefore, performing testing for AI during ongoing HD would facilitate the procedure [14, 15]. There are concerns about metabolism and clearance for adrenocorticotropic hormone and cortisol in addition to hemoconcentration due to removal of plasma water (ultrafiltration) as a confounding factor during HD. Some [16–18], but not all studies [19], report that free plasma cortisol is transferred into the dialysate during HD, which leads to a decrease in plasma cortisol concentration. Due to these concerns, the ACTH test is routinely performed before or between HD sessions. The ACTH test has not been especially validated in patients on HD and continuous ambulatory peritoneal dialysis (CAPD).

This review of the literature aims to evaluate the current evidence for validity of testing for AI in patients on chronic renal replacement therapy.

## Methods

### Search strategy and study selection

We intended to perform a systematic review. This article therefore adheres to the “Preferred Reporting Items for Systematic Reviews and Meta-Analyses” (PRISMA) guidelines and to the ethical standards of the Declaration of Helsinki. No ethical approval was necessary, as it is a systematic review of already published studies. A review protocol was generated and registrated at https://www.crd.york.ac.uk/prospero/ (registration no. CRD42020199245).

PubMed/Medline was used as the main source of information. The search has been performed since start of database to July 2020. Studies were identified using following keywords, crossed with connectors AND or OR: “ACTH testing”, “chronic renal replacement” (see Additional File for detailed search string). We reviewed bibliographies of reviewed articles and searched clinical trials for ongoing or unpublished trials. Two hundred fifteen potentially eligible publications were found with our systematic search. Additionally, three records were retrieved in the references of another one.

Eligibility assessment was performed by two reviewers (LB and CAB), and disagreements were solved by assessment through a third reviewer (MN). In a first step of exclusion, reviewers screened titles and abstracts for inclusion and exclusion criteria. Remaining articles were reviewed by full text screening. Inclusion criteria were human studies with adult patients on HD or CAPD undergoing testing for evaluation of AI. Exclusion criteria were: (1) not meeting inclusion criteria/ different subjects; (2) animal studies or in vitro studies; (3) case reports, reviews; (4) children under 18 years. There was no language restriction, restriction in publishing status or restriction in type of literature.

ACTH testing or similar testing for adrenal function in patients with HD were compared. The primary outcome was comparability of adrenal function testing to reference values. Furthermore, the mean difference between cortisol values was compared.

### Data collection process

Data was extracted by two reviewers. From each included article was the following information extracted: first author, year, country, study design, number of participants, sex, mean age, sort of renal replacement, type of renal disease, type of adrenal disease, performed ACTH test, timing of ACTH test, other test of adrenal function, outcome tested and effect size.

### Risk for bias assessment of individual studies and across studies

For assessing the risk of bias we used the “Scottish Intercollegiate Guidelines Network” (SIGN) system. The risk of bias was recorded using checklists and divided into “high”, “acceptable” and “unacceptable” quality (see Tables 1 and 2). Due to small selection, all 16 studies, regardless of quality, were used for further analysis.

**Table 1:** Risk of Bias Assessment of observational studies and case series

Author (Year) Study design	Appropriate question	Investigated factor is only difference between groups	Number of screened participant indicated	likelihood of outcome at time of enrolment is assessed	drop out before completed study [%]	comparison between full participants and those lost to follow up	clearly defined outcomes	Outcome assessment blinded to exposure status	exposure status may have influenced assessment of outcome	reliability of assessment of exposure	validity of outcome	> 1 assessment of exposure level or prognostic factor	identified confounders	provided confidence intervals	overall quality
Barbour GL (1974) Observational study [20]	Yes	n.a.	No	No	0	No	Yes	Can’t say	Yes	Yes	Yes	No	No	No	unacceptable
Deck KA (1979) [21] Observational study	Yes	n.a.	No	Can’t say	0	No	Yes	Can’t say	Yes	Can’t say	Yes	No	No	No	unacceptable
Vigna L (1995) Observational study [22]	Yes	Can’t say	No	Can’t say	0	No	Yes	Can’t say	Yes	Yes	Yes	Yes	Yes	No	acceptable
Tsubo T (1996) Case series [23]	Yes	n.a.	No	n.a.	0	No	Yes	n.a.	Yes	Yes	Yes	Yes	No	Yes	acceptable
Sakao Y (2014) Case series [24]	Yes	n.a.	Yes	n.a.	0	No	Yes	n.a.	Yes	Yes	Yes	Yes	Yes	No	acceptable
Koh TJK (2016) Case series [25]	No	n.a.	No	n.a.	0	No	Can’t say	n.a.	Yes	Yes	Yes	No	No	No	unacceptable

**Table 2:** Risk of Bias Assessment of case-control studies

Author (Year)	Appropriate, clearly focused question	Comparable cases and controls	Same exclusion criteria for cases and controls	Percentage of group participated	Comparison between participants and non-participants	Clearly defined cases	Controls are non-cases	Measures to prevent knowledge of primary exposure influencing case ascertainment	Exposure status are measured in standard, valid, reliable way	Main potential confounders are identified and taken into account in the design and analysis	Provided confidence intervals	Overall quality
Akmal M (1977) [26]	Yes	No	No	Cases: 100%, Controls: 100%	no	Yes	Yes	n.a.	Yes	Yes	No	Unacceptable
Ramirez G (1982) [27]	Yes	Can’t say	Can’t say	Cases: 100%, Controls: 100%	no	Yes	Yes	n.a.	Yes	Yes	Yes	Acceptable
Zager PG (1985) [28]	Yes	No	Can’t say	Cases: 100%, Controls: 100%	no	Yes	Yes	n.a.	Yes	Yes	Yes	Acceptable
Siamopoulos KC (1988) [29]	Yes	Yes	Can’t say	Cases: 100%, Controls: 100%	no	Yes	Yes	n.a.	Yes	Yes	No	Acceptable
Watschinger B (1991) [30]	Yes	Can’t say	Can’t say	Cases: 100%, Controls: 100%	no	Yes	Yes	n.a.	Yes	Yes	Yes	Acceptable
Grant AC (1993) [31]	Yes	Yes	Can’t say	Cases: 100%, Controls: 100%	no	Yes	Yes	n.a.	Yes	Yes	Yes	Acceptable
Clodi M (1998) [32]	Yes	Yes	Yes	Cases: 100%, Controls: 100%	no	Yes	Yes	n.a.	Yes	Yes	Yes	High quality
Oguz Y (2003) [33]	Yes	Yes	Yes	Cases: 100%, Controls: 100%	no	Yes	Yes	n.a.	Yes	Yes	Yes	High quality
Arregger AL (2014) [9]	Yes	Yes	Yes	Cases: 70.5%, Controls: 100%	no	Yes	Yes	n.a.	Yes	Yes	Yes	High quality
Valentin A (2020) [6]	Yes	Yes	Yes	Cases: 68.2%, Controls: 53.6% HD, 88.2% CAPD	no	Yes	Yes	n.a.	Yes	Yes	Yes	High quality

We also consulted the Cochrane Handbook for Systematic Reviews of Interventions.

### Data synthesis and statistical analysis

A minimum of five trials were required for the quantitative analysis (meta-analysis).

Dichotomous data was expressed as risk ratios (RR) with 95% Confidence Intervals (CI), continuous data as standard mean differences (SMD) with 95% CI. As a test of heterogeneity, the variation in SMD across studies attributable to heterogeneity (I^2^) was computed [34]. As there was significant heterogeneity across studies (I^2^ > 75%), data of the fixed-effect model was omitted due to overestimation of effect size. Data was pooled using a random effects model. For each study, the effect size was plotted by the inverse of its standard error [18]. The symmetry of these “funnel plots” were assessed both visually and formally with Egger’s test to see if the effect decreased with increasing sample size.

The statistical analysis was conducted using Stata software v15.1 (Stata Corp., College Station, TX, USA). All significance tests were two-sided, and *p*-value of < 0.05 was considered to be statistically significant.

## Results

**Fig. 1:**
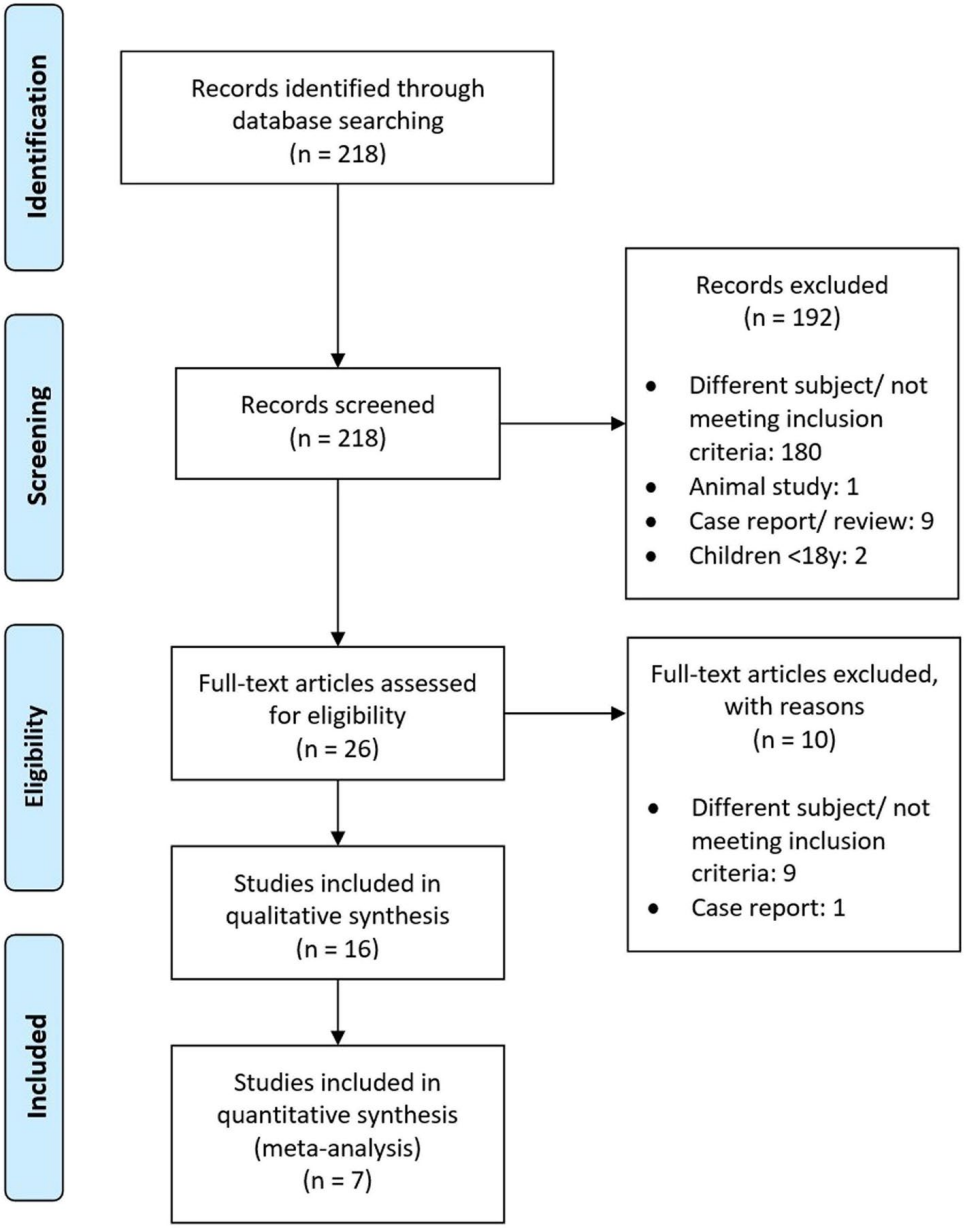
PRISMA flow chart of systematic search

After screening 218 identified titles and abstracts for exclusion criteria, 192 articles were excluded. The remaining 26 articles were reviewed by full text screening, whereby another 10 articles were ruled out. In total, 16 articles were eligible for the qualitative analysis. Of these, three were observational studies, three case series, and 10 case-control studies (see also Study Flow Chart in Fig. 1 and Additional Table 1 and Additional Table 2 in the Additional File).

The 16 identified studies included 381 participants (five to eighty). The studies were published between 1974 and 2019 in twelve different countries (see Table 3).

**Table 3:** Study Basics Part I

Author, Year	Country	ACTH test	Cosyntropin dose	baseline cortisol	measured ACTH	n (%) of detected AI	of which primary AI	other tests
Barbour GL, 1974 [20]	USA	Yes	25 U	Yes	No	0	0	
Akmal M, 1977 [26]	USA	Yes	250 μg i.m.	Yes	Yes	0	0	metyrapone test^a^, pre−/postHD serum cortisol & ACTH
Deck KA, 1979 [21]	Germany	No		Yes	No	0	0	
Ramirez G, 1982 [27]	USA	No		Yes	Yes	0	0	metyrapone test, dexamethasone suppression test, insulin-induced hypoglycemia/ insulin tolerance test, CRH test
Zager PG, 1985 [28]	USA	No		Yes	Yes	0	0	ACTH infusion after Dexamethason overnight before HD
Siamopoulos KC, 1988 [29]	Greece	No		Yes	Yes	0	0	CRH on a non-HD day
Watschinger B, 1991 [30]	Austria	No		Yes	Yes	0	0	CRH
Grant AC, 1993 [31]	UK	No		Yes	Yes	not specified	0	basal plasma ACTH precursors, CRH
Vigna L, 1995 [22]	Italy	Yes	250 μg	Yes	Yes	0	0	CRH
Tsubo T, 1996 [23]	Japan	No		Yes	No	0	0	cortisol at 2 h/12 h/24 h/48 h from start of CHDF; Epinephrin, Norepinephrin, Dopamine
Clodi M, 1998 [32]	Austria	Yes	1, 5, and 250 μg	Yes	Yes	0	0	
Oguz Y, 2003 [33]	Turkey	Yes^b^	250 μg	Yes	No	0	0	UFC, midnight-to-morning Cort/Cr increment, Dexamethason suppression test
Arregger AL, 2014 [9]	Argentina	No		Yes	No	10 (20%)	4 (40%)	basal saliva cortisol
Sakao Y, 2014 [24]	Japan	Yes	250 μg	Yes	Yes	5 (100%)	1 (20%)	CRH test
Koh TJK, 2016 [25]	Canada, Singapore	No		Yes	No	5 (100%)	0	
Valentin A, 2020 [6]	Denmark	Yes	250 μg	Yes	No	1 (3%)	0	
**Total**		7		16	9			

*ACTH* Adrenocorticotropic hormone, *AI* Adrenal insufficiency, *UFC* Urin free cortisol, *CRH* Corticotropin-releasing hormone, *HD* Hemodialysis, *CHDF* continuous hemodiafiltration, *Cort/Cr* UFC/creatinine ratio

^a^Metyrapone test detected steroid-induced AI in 2 patients

^b^Performed in all patients 1 day after last HD, but in 10 patients also on HD day

Whereas most studies gave information on the time of day, fasting was rarely mentioned, and the influence of the menstrual cycle or of oestrogens on test results in female subjects was infrequently considered (see also Additional Table 3 in the Additional File).

### Qualitative analysis

Whereas most studies had the aim of investigating the responsiveness of the hypothalamo-pituitary-adrenal axis in asymptomatic patients on HD, three studies tested for adrenal insufficiency due to clinical suspicion. The case series of Sakao et al. reported AI as a secondary cause for hypercalcemia [24]. Arregger et al. and Koh et al. performed adrenal function testing due to hypotension in HD patients [9, 25].

#### Pharmacokinetic aspects of serum cortisol, ACTH and CRH

Only Deck et al. investigated the change in cortisol with respect to HD. They measured the plasma clearance rate of radioactive cortisol in seven patients during and after HD. Plasma clearance rate was 30–63% higher during HD in five out of seven cases, resulting in decreased plasma cortisol during and increased plasma cortisol after HD. At the same time, the dialyzability of cortisol was low due to its high binding to transcortin. Therefore, the loss of cortisol in the dialysate hardly contributed to the change in plasma cortisol levels. Deck et al. were not sure about the exact cause of the increased plasma clearance rate of cortisol. An increased metabolism during HD due to increased protein binding or a change in the cortisol metabolism itself were discussed [21].

Akmal et al. also pointed out that the cortisol levels increased after the end of HD [26]. They assessed changes in cortisol pre- and post-HD in 21 HD patients and found that values post-HD about doubled as compared to pre-HD values. Furthermore, they compared serum cortisol and serum ACTH (both pre- and post-HD) in five patients without steroids and two patients who received large doses of steroid for about 6 weeks (no steroids during last 2 weeks before the test). After HD, there was an increase in cortisol and ACTH in all five non-steroid patients. The other two steroid patients had reduced values of ACTH and cortisol and were diagnosed with steroid-induced AI [26].

Tsubo et al. found that during continuous haemodiafiltration in intensive care patients, there were no significant changes in plasma cortisol levels [23].

Siamopoulos et al. investigated the kinetics of exogenous CRH in HD patients and patients with end-stage renal failure before initiation of renal replacement therapy. In patients without HD, CRH values were in upper normal range. They postulated that accumulated uremic toxins could inhibit the enzymes which degrade CRH. Therefore, clearance rate would be lower and CRH levels would increase. On the other hand, HD could benefit the degradation of CRH by eliminating these inhibiting toxins. CRH in HD patients was still in a normal, but lower range. They concluded that the kidney was not primarily responsible for the degradation of CRH [29].

#### ACTH testing

ACTH testing was performed in seven of the retrieved 16 studies, but in none of these during ongoing HD [6, 20, 22, 24, 26, 32, 33].

Sakao et al. and Valentin et al. both performed a stimulation test with 250 μg ACTH. The study from Valentin et al. was the only case-control study which allegedly detected adrenal insufficiency in HD patients based on lab value (insufficient response to ACTH, cutoff defined at 420 nmol/l) [6]. In their cohort of patients on HD not treated with prednisolone, only 3% of cases (1/30) had hypoadrenalism. In the case series of Sakao et al., all five participants (100%) obviously had an AI, of which four had secondary AI and one had primary AI due to unilateral adrenalectomy [24]. In the other five studies which performed an ACTH test, there were no HD patients diagnosed with AI. In the cases, cortisol increased significantly after ACTH stimulation, and there was no significant difference to controls [20, 22, 26, 32, 33].

Clodi et al. described the time of the peak of serum cortisol after stimulation with different doses of exogenous ACTH [32]. The test was performed in seven HD patients (before HD), seven CAPD patients, and seven healthy controls. On one hand, there was a significant increase in serum cortisol after stimulation in all three groups but with different peak times (30 min after 1 μg, 60 min after 5 μg and 120 min after 250 μg). On the other hand, they also described a trend towards blunted and delayed cortisol release following 1 μg ACTH in HD patients.

#### CRH stimulation test

Grant et al. and Vigna et al. both performed a stimulation test using 100 μg synthesized corticotropin releasing hormone (CRH) [22, 31]. They investigated the time of the peak of ACTH and cortisol after injection. The control groups in both studies reached the ACTH peak 30 min after CRH administration. While HD and CAPD patients in the study of Grant et al. also showed the peak of ACTH after 30 min, the peak of HD patients in Vigna et al. occurred later (after 60 min). There was no difference in time of cortisol peak between the case and control groups in each study, as well as between the two studies (Grant: 30 to 60 min, Vigna: 60 min).

Four other studies also conducted a CRH stimulation test with different results [24, 27, 29, 30]. Siamopoulos et al., Ramirez et al. and Vigna et al. all reported increased stimulated plasma cortisol in controls and cases [22, 27, 29]. While an increase of ACTH was shown in the study of Ramirez et al. [27], Siamopoulos’ study showed blunted values [29]. Watschinger et al. also showed a slightly blunted response of plasma ACTH to CRH [30]. Contrarily, in the case series of Sakao et al., peak levels of ACTH and cortisol were both lower than two-fold of their basal values in four out of five cases. These patients were therefore classified as having AI [24].

Zager et al. showed that the reaction of cortisol and other adrenal hormones after infusion of ACTH in CAPD patients was comparable to healthy subjects [28].

#### Other tests

Ramirez et al. performed an insulin tolerance test in nine HD patients (between HD) and thirteen healthy controls. Plasma glucose, cortisol and ACTH were measured. Patients with renal failure showed prolonged and sustained hypoglycemia with plasma cortisol remaining within the normal range. Contrarily, healthy controls showed a physiological increase in plasma cortisol due to hypoglycemia and then gradually returned to baseline levels. The same observation was made for ACTH plasma concentration. The reaction of plasma cortisol and plasma ACTH concentration was also tested using a metyrapone test. There was no difference in the increase of plasma ACTH or plasma ACTH values between HD patients and healthy controls. HD patients and controls had a reduction of plasma cortisol after stimulation. Post-metyrapone cortisol was higher in HD patients than controls but without statistical significance [27].

In contrast to the other studies, the control group of Valentin et al. did not consist of healthy people, but of kidney transplant patients receiving low-dose prednisolone treatment. It was the only study in which the control group had a higher incidence of secondary AI (43.3%) than HD patients (3.3%). This can be explained by the fact that kidney transplant patients were subjected to years of steroid therapy, which led to a secondary AI, while HD patients included in this study were not treated with systemic glucocorticoids [6].

### Quantitative analysis: baseline serum cortisol

For the quantitative meta-analysis, seven case-control studies with 210 patients had comparable data of baseline serum cortisol [9, 26, 27, 29, 31–33] (see Table 4). All selected studies had a control group for comparison. Cases were patients on HD. As there were only three studies giving comparable data on stimulated cortisol levels after ACTH testing, we did not perform a meta-analysis of stimulated cortisol There were too few studies to perform the same evaluation for CAPD patients.

**Table 4:** Study Basics Part II

Author (Year)	Study design	N total	N cases	N controls	female gender cases, n (%)	female gender controls, n (%)	mean age cases (range or SD)	mean age controls (range or SD)	Type of renal replacement
Barbour GL, 1974 [20]	observational study	7	7	0	0	n.a.	unknown	n.a.	HD
Akmal M, 1977 [26]	case-control study	17	11	6	5 (46%)	unknown	47.8	29.6	HD
Deck KA, 1979 [21]	observational study	7	7	0	2 (29%)	n.a.	28–38	n.a.	HD
Ramirez G, 1982 [27]	case-control study	20	10	10	0	0	51.5 (28–65)	40.4 (27–61)	HD
Ramirez G, 1982 [27]	case-control study	22	9	13	0	0	55.2 (38–65)	37 (25–61)	HD
Ramirez G, 1982 [27]	case-control study	10	5	5	0	0	48 (38–61)	53.6 (49–61)	HD
Zager PG, 1985 [28]	case-control study	13	6	7	3 (50%)	0	58.8 ± 9.8	23 ± 3.6	CAPD
Siamopoulos KC, 1988 [29]	case-control study	19	13	6	6 (46%)	3 (50%)	48.4 ± 10.4	46.2 ± 3.5	HD
Watschinger B, 1991 [30]	case-control study	15	7	8	0	0	22–43	unknown	HD
Grant AC, 1993 [31]	case-control study	30	20	10	10 (50%)	5 (50%)	46 (18–69)	42 (19–58)	10 HD, 10 CAPD
Vigna L, 1995 [22]	observational study	10	10	0	4 (40%)	n.a.	53 (22–71)	n.a.	HD
Tsubo T, 1996 [23]	case series	10	10	0	5 (50%)	n.a.	58.0 ± 3.3	n.a.	CHDF
Clodi M, 1998 [32]	case-control study	21	14	7	0	0	49.0 ± 6.2 HD, 43.5 ± 4.8 CAPD	39.6 ± 4.2	7 HD, 7 CAPD
Oguz Y, 2003 [33]	case-control study	30	16	14	0	0	35.19 ± 14.12	27.43 ± 10.34	HD
Arregger AL, 2014 [9]	case-control study	80	50	30	23 (46%)	16 (53%)	25–65	43.7 ± 8.8 (20–58)	48 HD, 2 CAPD
Sakao Y, 2014 [24]	case series	5	5	0	unknown	n.a.	69 ± 7	n.a.	HD
Koh TJK, 2016 [25]	case series	5	5	0	2 (40%)	n.a.	20–55	n.a.	HD
Valentin A, 2020 [6]	case-control study	60	30	30	11 (37%)	13 (43%)	59.0 ± 13.1	50.4 ± 13.1	15 HD, 15 CAPD
**Total**		381	235	146					

*HD* Hemodialysis, *CAPD* Continuous ambulatory peritoneal dialysis, *CHDF* Continuous hemodiafiltration

Controls were healthy subjects except Valentin et al. which were renal transplant patients

^1^pre−/post-HD serum cortisol in 14 patients, serum cortisol and adrenocorticotropic hormone (ACTH) (pre−/post-HD) on additional group of 7

^2^Two controls and 2 uraemic patients for volume distribution, 20 for 17-OHCS measurements

^3^Metyrapone test

^4^Insulin-induced Hypoglycemia

^5^Corticotropin Stimulation test

^6^Fifty-six patients with chronic kidney insufficiency were part of this study, but only 16 were on HD

**Fig. 2:**
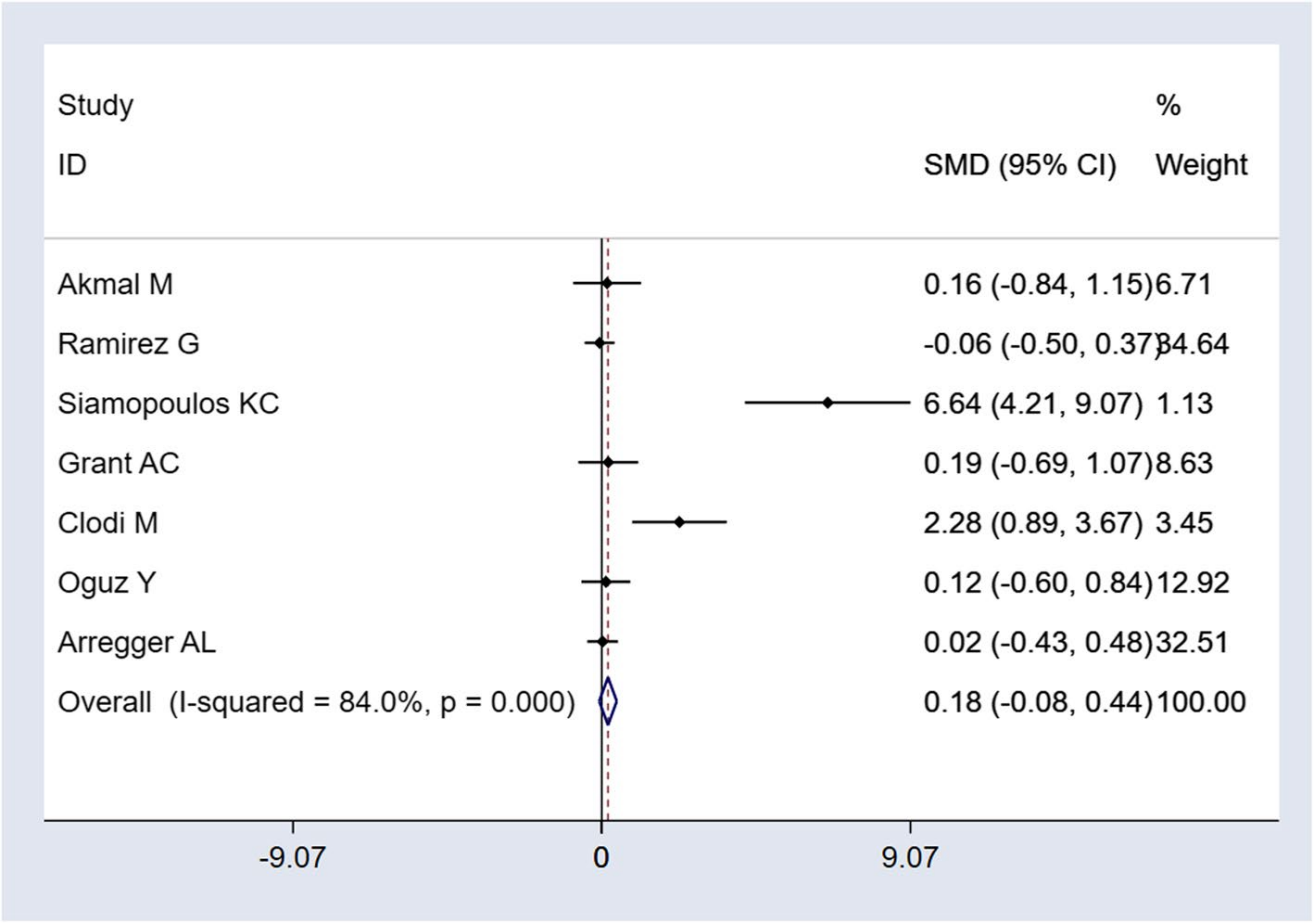
Mean basal cortisol levels of comparable studies

The meta-analysis showed no statistically significant difference in basal cortisol of HD patients compared to the control group. (SMD 0.18; 95% CI -0.08, 0.44; see Fig. 2).

### Risk of bias assessment

The risk of bias was considered in all 16 studies using the SIGN checklists. The overall quality of two out of three observational studies and one out of three case series was assessed as unacceptable (see also Tables 1 and 2). There was no high-quality study among them. Only one case-control study was considered unacceptable. The remaining nine case-control studies were of high quality or acceptable.

### Heterogeneity and publication bias

**Fig. 3:**
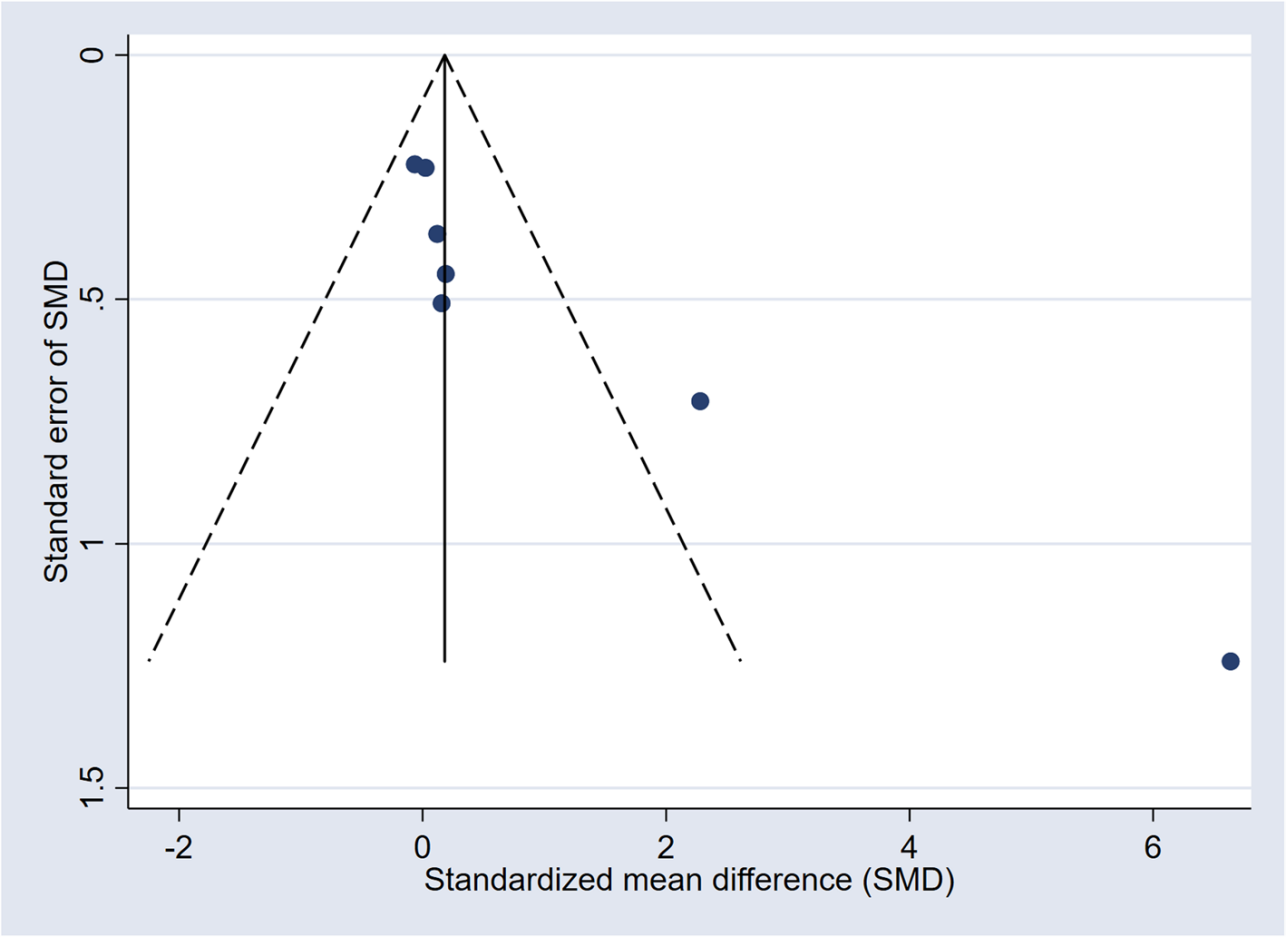
Funnel plot of basal cortisol, comparable studies

There was a high heterogeneity among the studies (I^2^ = 84%). The funnel plot for basal cortisol was symmetrical except two extreme outliers, thus showing heterogeneity. Formal testing by the Egger’s test refuted the H0 hypothesis of small study effects (*p* = 0.016) [35]. (See also Fig. 3).

## Discussion

To the best of our knowledge, this is the first attempt to generate a systematic review and meta-analysis on the validity of adrenal function testing in patients undergoing renal replacement therapy.

First, we found limited data available regarding tests of adrenal function during HD. All published studies have performed the tests before or in between HD sessions. Only Deck et al. dealt with the clearance of cortisol during HD. The study showed lower cortisol levels during HD as compared to after HD. This effect is most likely explained by the dialyzation of free cortisol [36], as compared to protein-bound cortisol, and there is evidence that this decrease in plasma cortisol leads to a counter-regulatory ACTH secretion and thus to an increase of cortisol production during the next four hours [16–18].

Second, there is little data on ACTH testing before HD or on a non-HD day [6, 20, 22, 24, 26, 32, 33]. ACTH - stimulated cortisol significantly increased in most studies. But there was a trend to blunted and delayed cortisol responses after low-dose, i.e. 1 μg, ACTH testing in HD patients.

Third, all sixteen studies had basal cortisol levels, but only some of them also measured basal ACTH. The basal cortisol was used more often for evaluation of an intact hypothalamo-pituitary-adrenal axis in patients on renal replacement therapy, despite its inherent limitations for interpretation.

The meta-analysis of baseline serum cortisol levels revealed no significant difference between cases and controls regarding basal serum cortisol, showing that basal cortisol values are comparable to reference values in patients undergoing HD. There was, however, a significant heterogeneity of studies, differing sometimes greatly in terms of study population regarding the age and gender of the participants, type of renal disease, the HD duration since start, etc. Vigna et al. justified the differing results of several studies with these confounders [22].

A major limitation is that the current data shows comparability of cortisol to reference values and feasibility of ACTH testing in asymptomatic patients undergoing HD without clinical suspicion of AI, but not of true cases of AI, be it primary or secondary AI, except in three case series. Therefore, there remains very limited availability of data on the validity of ACTH testing for diagnosing adrenal insufficiency in patients on HD.

If confirming or excluding adrenal insufficiency in patients on HD is necessary, current evidence suggests measuring a serum morning cortisol in patients on HD may be useful, as serum cortisol values are comparable in patients on HD to healthy volunteers. Current guidelines for diagnosing AI in general [1] suggest that very low values < 80 nmol/l or values of > 500 nmol/l may already be considered sufficient to diagnose or rule out AI. In case of a serum cortisol level of < 80 nmol/l, performing an ACTH test to confirm the diagnosis of AI should be done to have sufficient rationale for permanent glucocorticoid replacement. If serum morning cortisol value is < 500 nmol/l, thus in most patients, an ACTH test should be performed according to general standards, i.e. preferentially in the morning due to the circadian cortisol rhythm [1]. Further studies are necessary to confirm that the generally used cut-off values of the ACTH test for diagnosing and ruling out AI are also valid for patients on HD.

Due to the pharmacokinetic evidence and lack of clinical studies, ACTH testing should be performed either before HD or on a day without HD. Theoretically, postponing the test from before HD to during HD would benefit the patient in terms of comfort. The ACTH test takes over 1 hour, which the patient must spend in the hospital in addition to his time on HD. However, results of Deck et al. regarding plasma clearance rate indicate that false low cortisol levels may occur during HD, thus potentially leading to an overdiagnosing of AI [21]. Further studies are required to specifically verify the reliability of an ACTH test or other tests during HD.

## Conclusions

In patients undergoing renal replacement therapy, basal serum cortisol values are comparable to healthy volunteers. There is limited data on the validity of stimulated cortisol in these patients, especially during HD.

## Supplementary Information

**Additional file 1: Additional Table 1**. Reasons for first step of exclusion (titles and abstracts). **Additional Table 2**. Reasons for second step of exclusion (full text). **Additional Table 3**. Additional baseline information with relation to testing.

## Data Availability

The data underlying this article are available in the article and in its online supplementary material.

## Abbreviations

ACTH: Adrenocorticotropic hormone
AI: Adrenal insufficiency
CAPD: Continuous ambulatory peritoneal dialysis
CI: Confidence interval
CRH: Corticotropin releasing hormone
HD: Hemodialysis
PRISMA: Preferred Reporting Items for Systematic Reviews and Meta-Analyses
RR: Risk ratio
SIGN: Scottish Intercollegiate Guidelines Network
SMD: Standard mean differences
