# Supplementary material for: Adrenal function testing in dialysis patients – a review of the literature
Source: BMC Nephrol. 2021 Nov 1;22:360. doi: 10.1186/s12882-021-02541-5 (PMC8561863; doi:10.1186/s12882-021-02541-5)
Supplement: Supplementary file 1 — Additional file 1: Additional Table 1. Reasons for first step of exclusion (titles and abstracts). Additional Table 2. Reasons for second step of exclusion (full text). Additional Table 3. Additional baseline information with relation to testing. [file 12882_2021_2541_MOESM1_ESM.docx]

**Additional File**

**to**

Adrenal function testing in dialysis patients

– a systematic review and meta-analysis

**PubMed complete search string:**

((("Cosyntropin"[Mesh]) OR ("Adrenal Insufficiency"[Mesh]) OR ("Addison Disease"[Mesh]) OR (("Hydrocortisone"[Mesh]) AND ("Kinetics"[Mesh]))) AND (("Renal Dialysis"[Mesh]) OR ("Renal Replacement Therapy"[Mesh]))) OR ((synacthen OR cosyntropin OR tetracosactide OR "adrenal insufficiency" OR addison OR (cortisol AND kinetic*) OR "ACTH test") AND (dialysis OR "renal replacement therapy" OR hemodialysis OR "peritoneal dialysis")

**Additional Table 1. Reasons for Step 1 of exclusion (titles and abstracts)**

| **Reasons for step 1 of exclusion (title and abstract)** | **Numbers: of 215** |
| --- | --- |
| **From cross-references** | **3** |
|  | **Of 218** |
| Not meeting inclusion criteria/different subject | 180 |
| Animal study | 1 |
| Case report/correspondence/review | 9 |
| Children <18 y | 2 |
| Identified for full-text evaluation | 26 |

**Additional Table 2. Reasons for Step 2 of exclusion (full text)**

| **Reasons for step 2 of exclusion (full text)** | **Numbers: of 26** |
| --- | --- |
| Not meeting inclusion criteria/different subject | 9 |
| Animal study | 0 |
| Case report/correspondence/review | 1 |
| Children <18 y | 0 |
| Identified for inclusion in systematic review | 16 |

**Additional Table 3. Additional baseline information with relation to testing**

| **Author (year)** | **Time of day** | **Fasting** | **Consideration of menstrual cycle/oestrogens** |
| --- | --- | --- | --- |
| Barbour GL 1974^1^ | 8 a.m. | Not mentioned | Male only |
| Akmal M 1977^2^ | 9 a.m. | Not mentioned | Not mentioned |
| Deck KA 1979 ^3^ | Not mentioned | Not mentioned | Not mentioned |
| Ramirez G 1982^4^ | 8 a.m. | Not mentioned | Male only |
| Zager PG 1985^5^ | 8 a.m. | Not mentioned | Not mentioned |
| Siamopoulos KC 1988^6^ | 8 a.m. | Not mentioned | Not mentioned |
| Watschinger B 1991^7^ | 6 p.m. | Not mentioned | Not mentioned |
| Grant AC 1993^8^ | 2 p.m. | Not mentioned | Not mentioned |
| Vigna L 1995^9^ | 8h30 a.m. | Yes | No medication interfering with HPA axis |
| Tsubo T^10^ 1996 | Not mentioned | Not mentioned | Not mentioned |
| Clodi M 1998^11^ | 5 p.m. | 4h fast | Male only |
| Oguz Y 2003^12^ | Between 7 and 8 a.m. | Not mentioned | Male only |
| Arregger AL 2014^13^ | Between 8 and 9 a.m. | Not mentioned | Not mentioned |
| Sakao Y 2014^14^ | Early morning | Yes | One female only, postmenopausal |
| Koh TJK 2016^15^ | Random or morning | Not mentioned | Not mentioned |
| Valentin A 2020^16^ | morning | Yes | 8 weeks of oestrogen pause |

**References**

1 Barbour, G. L. & Sevier, B. R. Letter: Adrenal responsiveness in chronic hemodialysis. *N Engl J Med* **290**, 1258, doi:10.1056/NEJM197405302902213 (1974).

2 Akmal, M. & Manzler, A. D. Simplified assessment of pituitary-adrenal axis in a stable group of chronic hemodialysis patients. *Trans Am Soc Artif Intern Organs* **23**, 703-706, doi:10.1097/00002480-197700230-00190 (1977).

3 Deck, K. A., Fischer, B. & Hillen, H. Studies on cortisol metabolism during haemodialysis in man. *Eur J Clin Invest* **9**, 203-207, doi:10.1111/j.1365-2362.1979.tb00924.x (1979).

4 Ramirez, G., Gomez-Sanchez, C., Meikle, W. A. & Jubiz, W. Evaluation of the hypothalamic hypophyseal adrenal axis in patients receiving long-term hemodialysis. *Arch Intern Med* **142**, 1448-1452 (1982).

5 Zager, P. G., Spalding, C. T., Frey, H. J. & Brittenham, M. C. Low dose adrenocorticotropin infusion in continuous ambulatory peritoneal dialysis patients. *J Clin Endocrinol Metab* **61**, 1205-1210, doi:10.1210/jcem-61-6-1205 (1985).

6 Siamopoulos, K. C., Eleftheriades, E. G., Pappas, M., Sferopoulos, G. & Tsolas, O. Ovine corticotropin-releasing hormone stimulation test in patients with chronic renal failure: pharmacokinetic properties, and plasma adrenocorticotropic hormone and serum cortisol responses. *Horm Res* **30**, 17-21, doi:10.1159/000181019 (1988).

7 Watschinger, B. *et al.* Effect of recombinant human erythropoietin on anterior pituitary function in patients on chronic hemodialysis. *Horm Res* **36**, 22-26, doi:10.1159/000182100 (1991).

8 Grant, A. C. *et al.* Hypothalamo-pituitary-adrenal axis in uraemia: evidence for primary adrenal dysfunction? *Nephrology, dialysis, transplantation : official publication of the European Dialysis and Transplant Association - European Renal Association* **8**, 307-310 (1993).

9 Vigna, L. *et al.* The impact of long-term hemodialysis on pituitary-adrenocortical function. *Ren Fail* **17**, 629-637, doi:10.3109/08860229509037629 (1995).

10 Tsubo, T., Hashimoto, Y., Araki, I., Ishihara, H. & Matsuki, A. Cortisol and catecholamine kinetics during continuous hemodiafiltration in patients with multiple organ dysfunction syndrome. *Intensive Care Med* **22**, 1176-1178, doi:10.1007/BF01709332 (1996).

11 Clodi, M. *et al.* Adrenal function in patients with chronic renal failure. *American journal of kidney diseases : the official journal of the National Kidney Foundation* **32**, 52-55, doi:10.1053/ajkd.1998.v32.pm9669424 (1998).

12 Oguz, Y. *et al.* The midnight-to-morning urinary cortisol increment method is not reliable for the assessment of hypothalamic-pituitary-adrenal insufficiency in patients with end-stage kidney disease. *J Endocrinol Invest* **26**, 609-615, doi:10.1007/BF03347016 (2003).

13 Arregger, A. L. *et al.* Adrenocortical function in hypotensive patients with end stage renal disease. *Steroids* **84**, 57-63, doi:10.1016/j.steroids.2014.03.008 (2014).

14 Sakao, Y. *et al.* Clinical manifestation of hypercalcemia caused by adrenal insufficiency in hemodialysis patients: a case-series study. *Intern Med* **53**, 1485-1490, doi:10.2169/internalmedicine.53.1104 (2014).

15 Koh, T. J. & Chan, C. T. Adrenal insufficiency presenting as unexplained hypotension in nocturnal home hemodialysis. *Hemodial Int* **20**, E10-13, doi:10.1111/hdi.12412 (2016).

16 Valentin, A. *et al.* Adrenal insufficiency in kidney transplant patients during low-dose prednisolone therapy: a cross-sectional case-control study. *Nephrology, dialysis, transplantation : official publication of the European Dialysis and Transplant Association - European Renal Association* **35**, 2191-2197, doi:10.1093/ndt/gfz180 (2020).
